# Continuity of care as a predictor of ongoing frequent attendance in primary care: a retrospective cohort study

**DOI:** 10.3399/bjgpopen20X101083

**Published:** 2020-10-14

**Authors:** Adam McDermott, Emily Sanderson, Christopher Metcalfe, Rebecca Barnes, Clare Thomas, Helen Cramer, David Kessler

**Affiliations:** 1 Centre for Academic Primary Care, Population Health Sciences, Bristol Medical School, University of Bristol, Bristol, UK; 2 Bristol Randomised Trials Collaboration, Bristol Medical School, University of Bristol, Bristol, UK

**Keywords:** General practice, continuity of care, frequent attenders, retrospective studies, primary health care

## Abstract

**Background:**

Frequent attenders (FAs) in primary care receive considerable resources with uncertain benefit. Only some FAs attend persistently. Modestly successful models have been built to predict persistent attendance. Nevertheless, an association between relational continuity of care and persistent frequent attendance remains unclear, and could be important considering both the UK government and Royal College of General Practitioner’s (RCGP) aim of improving continuity.

**Aim:**

To identify predictive measures (including continuity) for persistent frequent attendance that may be modified in future interventions.

**Design & setting:**

This is a retrospective cohort study sampling 35 926 adult patients registered in seven Bristol practices.

**Method:**

The top 3% (1227) of patients by frequency of GP consultations over 6 months were classed as FAs. Individual relational continuity was measured over the same period using the Usual Provider Continuity (UPC) index. Attendance change was calculated for the following 6 months. Multivariable logistic regression analysis was used to determine variables that predicted attendance change.

**Results:**

FAs were on average 8.41 years older (difference 95% confidence interval [CI] = 7.33 to 9.50, *P*<0.001) and more likely to be female (65.36% versus 57.88%) than non-FAs. In total, 79.30% of FAs decreased attendance over the subsequent 6 months. No association was found between continuity and subsequent attendance. Increasing age was associated with maintained frequent attendance.

**Conclusion:**

Continuity does not predict change in frequent attendance. In addition to improving continuity, recent government policy is focused on increasing primary care access. If both aims are achieved it will be interesting to observe any effect on frequent attendance.

## How this fits in

FAs are prevalent in general practice. Some FAs persistently have high attendance, while other FAs return to average levels of attendance over time. Good relational continuity of care in general practice has been associated with many positive outcomes. Despite this, previous models for predicting persistent frequent attendance have not explored any associations between relational continuity of care and persistent frequent attendance. This study has found that continuity of care within this sample of FAs did not predict subsequent change in attendance.

## Introduction

The reasons for patients attending their general practice frequently are varied, involving a complex mixture of physical and psychological issues.^1,2^ Frequent attendance may be appropriate, but may also lead to a poor use of resources with no increased patient benefit.^2,3^ As the top 3% of primary care attenders typically provide 14% to 15% of the workload, small reductions in the consultation rates of those FAs, whose GPs feel their high attendance is less appropriate, would lead to a significant reduction in workload.^1,2^ This could also reduce resource usage as, in 1991, UK FAs generated five times as many prescriptions and hospital contacts as average attenders.^3^ Additionally, between 2007 and 2009, the costs of primary care FAs in the Netherlands were triple that of average attenders.^4^ Despite this increased time and expenditure, FAs have been shown to generally have the same patient satisfaction scores as non-FAs.^3^

The UK government has aimed to improve continuity of care with the introduction of the named GP system.^5^ The *General Practice Forward View* states that providing continuity of care should be a core role of GPs.^6^ The RCGP also supports this aim through publishing a continuity toolkit and working with the Health Foundation in its 2019 funding programme for projects that improve continuity.^7,8^ Most studies regarding relational continuity of care (seeing the same clinician across several appointments) have been observational. Continuity has been associated with positive outcomes, including lower mortality, decreased admissions, increased medication adherence, and improved patient satisfaction and trust.^9–14^ Only two studies have assessed continuity in the FA population. The first collected data in the 1990s and was a snapshot of the levels of continuity in the FA population.^15^ The second study found that FAs with higher continuity experienced fewer hospital admissions.^10^ Modestly successful models have been built to predict persistent attendance using many variables although continuity has never been considered in their construction.^16^ Therefore, this study is the first to explore continuity and any association with the consultation rate of FAs over time.

The aim of this study was to present the characteristics of primary care FAs identified over a 6-month period and to identify predictive measures (including relational continuity of care) for ongoing persistent frequent attendance. These predictors could potentially be modified in future interventions.

## Method

### Setting

This retrospective cohort study was based on anonymised practice record data from seven practices, which were recruited via the Clinical Research Network: West of England for a feasibility trial of a primary care intervention to improve the care of frequent attenders.^17^

Practices ranged in size (from 7924 to 16 702 patients), deprivation (from the most to the least deprived national deciles), and setting (urban and semi-rural).^18^ None of the practices operated personal lists, micro-teams, or were undergoing specific continuity of care interventions at the time of study. All practices used 10-minute appointment slots.

Following ethical approval, as part of baseline data collection for the trial, all practices were asked to perform an EMIS search of GP–patient consultations over 12 months. Depending on when each practice undertook this search, these 12 month periods began between June and September 2014. Each consultation in the database listed patient ID, sex, age, and the GP seen.

### Patients

The searches were performed by practice staff and all patient data was fully anonymised, with only EMIS numbers available to the research team. Only patients aged >18 years and who had attended at least one consultation with a GP were included.

### Outcomes

FAs were defined as the top 3% of attenders within their own practice over a 6-month period. This definition has been used in previous literature, particularly with regards to continuity.^1,15^ One particular study comparing different FA definitions found that by classing FAs as a percentage of *‘* *top consulting patients* *’*, a distinct demographic group that was older and more female could be identified from the general population.^1^ Multiple studies have found that the smaller this ‘*top percentage of attenders*’ was defined, the stronger the association with specific demographics became.^1,19^ Initially the 3% threshold in the present study acted as an arbitrary cut-off, with some patients falling on either side despite having the same total consultations. Subsequently, this 3% cut-off was ‘rounded up’ to ensure that the FAs at the cut-off had at least one more consultation than the non-FAs beneath the cut-off. The authors used this percentage definition within each practice rather than the overall sample to account for the variations between practice characteristics, such as the use of different booking systems.

This study compared the FA and non-FA groups by demographics, mean consultations, mean age, sex, and mean consultations in each sex group. Demographics were also compared between practices in addition to: list size, number of GPs, and the National Deprivation Index (NDI) of the area served.^18^

**Figure 1.**
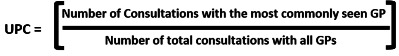
Calculation of Usual Provider Continuity index

Relational continuity in the first 6-month period of the study was calculated for each FA. This was done using the UPC index as recommended by the RCGP when measuring continuity at practices that do not use personal lists.^7^ This is defined as the proportion of consultations a patient has with their most commonly seen clinician, and is calculated as detailed in Figure 1. This index is frequently used in continuity of care research, and is easily calculated and interpreted.^20,21^ It is correlated with other measures of continuity such as the Continuity of Care indices.^22^

**Figure 2.**
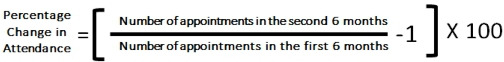
Calculation of percentage change in attendance

The study then explored any relationships between FA variables — including consultation rate in the initial 6 months, UPC index in the initial 6 months, age, sex, and practice — to any percentage change in attendance in the following 6 months (calculation is detailed in Figure 2).

### Analysis

This study employed Stata (version 14). When appropriate, simple two sample *t*-tests were used to compare demographic differences between the FA and non-FA groups.

The FA sample was analysed using a single multivariate linear regression model (with bias-corrected and accelerated bootstrapped 95% CIs). This model used each patient’s initial consultation rate, UPC index, age, sex, and their registered practice as variables for predicting subsequent changes in attendance. Bootstrapped CIs were used to avoid unreasonable assumptions concerning the sampling distributions.^23^ This method of analysis has been used in previous studies.^16,24^ All included variable data were complete. Social class and ethnicity were excluded from the study due to large amounts of missing data.

## Results

### Sample

There were 35 926 patients in this sample, of which 1227 patients accounted for the top 3% of attenders at their practice over the initial 6 months. These 1227 patients were therefore classed as FAs. Registered patients who did not attend at least once in this study period were excluded.

### Demographics of sample

**Figure 3.**
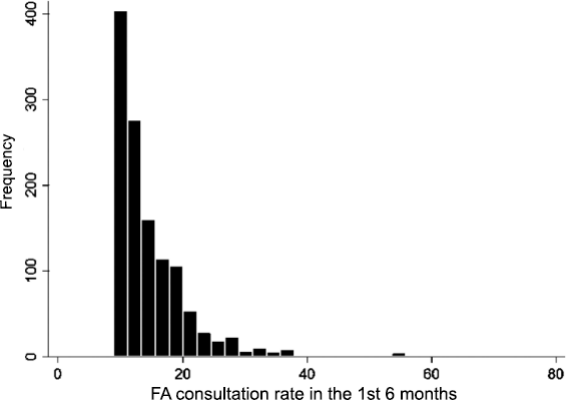
Distribution of 'number of consultations' in the first 6 months against 'patient frequency'.

Most patients in the sample attended only once (*n* = 12 436) or twice (*n* = 7641), while FAs within this sample consulted 15.13 times on average over the first 6 months, with a range of 9 to 76 consultations (Figure 3).

The number of consultations that qualified a patient as an FA varied across practices from 9 to 16 consultations in 6 months. FAs accounted for 15.81% of the consultations within this sample.

When compared to non-FAs, FAs were on average 8.41 years older (95% CI = 7.33 to 9.50, *P*<0.001) and more likely to be female (65.36% compared to 57.88%). These observations are consistent across all practices, with the exception of Practice 1, where the balance of sexes within the FA group was approximately equal.

Despite the proportion of females being higher in the FA group than the non-FA group, female FAs consulted no more than male FAs (15.23 versus 14.96, a difference of 0.27 consultations with a 95% CI of -0.55 to 1.09, *P* = 0.26). For more details see Table 1.

**Table 1.** Practice and demographic variables

**Variables**	**Total sample**	**Practice 1**	**Practice 2**	**Practice 3**	**Practice 4**	**Practice 5**	**Practice 6**	**Practice 7**
**Patients registered, *n***	85 725	7924	13 444	13 602	16 702	8342	15 743	9968
**Patients in sample, *n***	35 926	3541	6491	5816	6315	3041	5860	4862
NFA	34 699	3419	6285	5592	6102	2943	5645	4713
FA	1227	122	206	224	213	98	215	149
**GPs in sample, *n***	117	11	21	12	19	15	20	19
**Top** **3** **%** **cut off point**								
**A**ppointments/6 months, *n*****	NA	≥10	≥10	≥11	≥9	≥13	≥10	≥16
**Mean consultation rate over first 6 months (SD**)	3.27 (±3.38)	3.04 (±2.99)	2.94 (±2.67)	3.39 (±3.27)	2.74 (±2.94)	3.83 (±3.7)	2.97 (±2.96)	4.42 (±4.79)
NFA	2.85 (±2.22)	2.64 (±1.93)	2.62 (±1.91)	2.93 (±2.14)	2.37 (±1.7)	3.38 (±2.53)	2.57 (±1.93)	3.82 (±3.09)
FA	15.13 (±6.97)	14.25 (±5.11)	12.64 (±4.13)	14.72 (±5.39)	13.40 (±7.56)	17.42 (±6.52)	13.51 (±5.01)	23.24 (±8.95)
**Mean age, years (SD**)	50.29 (±19.12)	53.13 (±20.2)	55.62 (±19.01)	50.28 (±19.22)	48.52 (±18.26)	45.56 (±17.37)	47.76 (±18.45)	49.42 (±19.41)
NFA	50.00 (±19.98)	52.89 (±20.1)	55.30 (±18.85)	50.01 (±19.12)	48.05 (±18)	45.33 (±17.25)	47.62 (±18.41)	49.11 (±19.25)
FA	58.41 (±21.12)	59.80 (±21.81)	65.36 (±21.36)	56.82 (±20.61)	61.92 (±20.3)	52.41 (±19.17)	51.52 (±19.17)	58.97 (±21.88)
**Sex, female (%**)	58.13	60.15	58.07	57.60	56.06	57.94	59.51	58.56
NFA	57.88	60.16	57.69	57.42	55.87	57.42	59.22	58.29
FA	65.36	59.84	69.42	62.05	61.50	73.47	66.98	67.11
**NFA mean consultation rate over the first 6 months (SD**)	2.85 (±2.22)	2.64 (±1.93)	2.62 (±1.91)	2.93 (±2.14)	2.37 (±1.70)	3.38 (±2.53)	2.57 (±1.93)	3.82 (±3.09)
Male	2.65 (±2.1)	2.43 (±1.83)	2.46 (±1.85)	2.74 (±2.07)	2.32 (±1.61)	3.19 (±2.43)	2.43 (±1.89)	3.41 (±2.85)
Female	3.00 (±2.29)	2.79 (±1.98)	2.74 (±1.95)	3.07 (±2.18)	2.48 (±1.75)	3.52 (±2.6)	2.66 (±1.95)	4.12 (±3.22)
**FA mean consultation rate over the first 6 months (SD**)	15.13 (±6.97)	14.25 (±5.11)	12.64 (±4.13)	14.72 (±5.39)	13.40 (±7.56)	17.42 (±6.52)	13.51 (±5.01)	23.24 (±8.95)
Male	14.96 (±6.56)	14.43 (±5.14)	12.19 (±2.48)	14.05 (±5.46)	13.99 (±6.8)	18.65 (±8.99)	13.34 (±3.97)	22.63 (±8.13)
Female	15.23 (±7.18)	14.14 (±5.13)	12.83 (±4.7)	15.14 (±5.33)	13.03 (±8)	16.97 (±5.37)	13.59 (±5.46)	23.55 (±9.35)
**Mean UPC in the first 6 months for FAs (SD**)	0.57 (±0.22)	0.65 (±0.21)	0.66 (±0.21)	0.64 (±0.23)	0.52 (±0.22)	0.40 (±0.15)	0.49 (±0.2)	0.53 (±0.19)
**Practice area IMD decile^a^**	NA	4th	10th	5th	9th	1st	6th	3rd

^a^1st decile = more deprived; 10th decile = least deprived. FA = frequent attender. IMD = Index of Multiple Deprivation. NFA = non-frequent attender. SD = standard deviation. UPC = Usual Provider Continuity index.

### Frequent attenders and continuity of care

The FA group had a mean UPC index of 0.57 (standard deviation = ±0.22) suggesting that on average just over half of an FA’s appointments were with the same GP.

### Factors associated with change in rates of attendance

**Figure 4.**
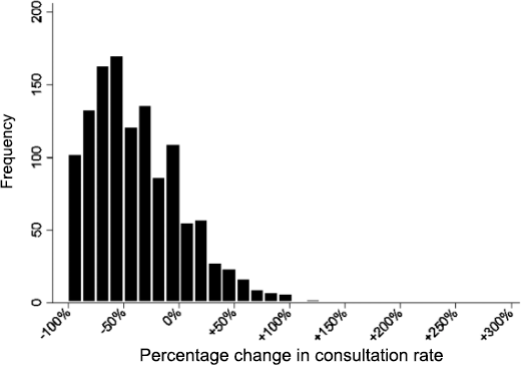
Distribution of 'percentage change in consultation rate' against 'patient frequency' for the frequent attender group

Of FAs, 79.30% decreased their attendance over the following 6 months; 20.70% of FAs maintained or increased their attendance compared to the previous 6 months (Figure 4).

For FAs in the first 6 months, this study used the previously described model to explore factors possibly associated with a change in their subsequent attendance, as displayed in Table 2.

**Table 2.** Multivariate model of predictors of percentage change in consultation rate among frequent attenders (*N* = 1227)

**Predictors**	**Coefficient, SD (95% CI**)	***P* value**
Consultation rate in the first 6 months	0.003 (-0.000 to -0.007)	0.105
Continuity of care in the first 6 months, UPC	-0.020 (-0.149 to -0.109)	0.772
Age (per 5 year increase)	0.01 (0.004 to -0.015)	0.001
Sex, female	0.042 (-0.008 to -0.094)	0.115
**Practice (** **m** **ean** **% change in attendance, SD), IMD decile,^a^ number of GPs**		
2 (-41.04, ±39.62), 10^th^, 21	Ref	Ref
4 (-42.92, ±40.93), 9^th^, 19	-0.013 (-0.097 to 0.065)	
6 (-33.86, ±43.39), 6^th^, 20	0.094 (0.01 to 0.179)	
3 (-28.47, ±50.10), 5^th^, 12	0.139 (0.042 to 0.224)	
1 (-48.24, ±38.96), 4^th^, 11	-0.062 (-0.142 to 0.034)	<0.001
7 (-28.71, ±40.78), 3^rd^, 19	0.103 (0.014 to 0.20)	
5 (-33.8%, ±42.44), 1^st^, 15	0.077 (-0.175 to 0.188)	

^a^1st decile = more deprived; 10th decile = least deprived. IMD = Index of Multiple Deprivation. UPC = Usual Provider Continuity.

This model showed that there was no association between UPC index in the first 6 months and any change of consultation rate over the next 6 months. For each additional 5 years in age, an FA’s subsequent consultation rate was predicted to be 1% higher than otherwise expected (coefficient = 0.01, 95% CI = 0.004 to 0.015, *P* = 0.001). There was also variation across the seven practices in regard to subsequent change in attendance. This practice variation did not have a relationship with practice deprivation deciles or the number of GPs in each practice. No significant associations with change in attendance were found for consultation rate over the first 6 months or sex. Details are displayed in Table 2.

## Discussion

### Summary

Within the FA group, varying continuity levels, as measured by UPC index over an initial 6-month period, did not predict rates of attendance in the ensuing 6 months. A positive but weak association was found between increasing age and increasing attendance in the FA group. This may not be important due to the small size of the difference and the relatively short study duration.

### Strengths and limitations

The main strength of this study is the large sample size, allowing the authors to detect differences in age and sex rates between the FA and non-FA groups. By collecting data from seven practices situated in areas with varying levels of deprivation, this study’s conclusions are more generalisable. In calculating the UPC index, the number of GPs in each practice were included, including doctors in training and temporary doctors. The most commonly seen GP was not necessarily a patient’s ‘named GP’. The authors feel that this is a strength in this study as it is a realistic picture of modern primary care, given that practices employ partners, salaried doctors, part-time doctors, trainees, and long-term/short-term locums. Tammes *et al* found that the introduction of the ‘named GP system’ has not led to an improvement in continuity.^25^ This contrasts with studies showing stronger continuity in practices operating personal lists, perhaps reflecting that for many practices the implementation of the ‘named GP system’ has been an administrative rather than a clinical exercise.^26,27^

Many studies have chosen the binary outcome of ‘still a frequent attender’ versus ‘no longer a frequent attender’.^16,24,28^ The authors decided to use a continuous outcome — percentage change in attendance — as this was not only more sensitive, but also more useful when considering clinical workload. Also, in contrast to many previous FA studies, the authors split their sample into two concurrent time periods enabling them to move beyond a cross-sectional ‘snapshot’ and look at frequent attendance longitudinally. Although this study’s measure of continuity, UPC, is limited in that it will overestimate continuity until a patient has had at least three consultations, this study has applied the UPC calculation only to FAs.^7^ Therefore this is not a limitation applicable to this analysis.

Limitations of this study include the difficulty extracting complete information from the GP records. Past literature has shown that some FAs consult due to psychosocial complaints, chronic illnesses, and medically unexplained symptoms.^28^ It would have been interesting to explore the reasons for consultation and to identify subgroups based on this information, but these data were unavailable. Some telephone consultations may have been followed by a face-to-face consultation, and hence recorded as two separate consultations potentially inflating some figures. Other limitations include having no access to the number of consultations with other allied healthcare professionals or whether a patient left the list during the study.

A further limitation is the relatively short study duration. Although the authors acknowledge that strong doctor–patient relationships can take a long time to form, this research explores FAs who consulted an average of 15 times over 6 months.^29^ Potentially, these patients would be able to form relationships within the period of the study and may have already had a relationship with their GP.

Regression to the mean contributed to a reduced number of consultations over the follow-up period. As the analysis was based on comparing risk groups among the FA, the association between those risk groups and subsequent frequency of consultation was separated from the effects of regression to the mean, as each risk group was subject to a similar degree of regression. In addition, the baseline number of consultations was included in multivariable models, and this covariate showed no association with percentage change in attendance.

### Comparison with existing literature

To the authors' knowledge, this is the first study to look at variations in continuity within the FA group and their association with ongoing attendance in primary care.

This study's demographic findings echo the literature in showing that FAs are older and more likely to be female.^30^ Furthermore, the authors found that the majority of FAs only temporarily consulted frequently.^16,24,28^ Previous studies have, however, indicated that neither sex nor age (as adjusted variables) were predictive of persistent frequent attendance.^16,24^

Consultation rates are higher in areas of increased deprivation.^31^ This study's model contained practices that spanned the national deciles.^17^ The practices that had the highest rate of attendance overall were in the first, third, and fifth deciles. Due to the sample size of seven practices, the authors cannot comment on associations between deprivation and ongoing frequent attendance.

### Implications for practice

There was no association between continuity and reduced attendance in the FA group in this study. It is possible that continuity could be associated with both increased dependence in some patients and increased enablement in others. Encouraging FAs to see a particular GP may not always be beneficial or even appropriate. Although it is accepted that the benefits outweigh the risks, high continuity has also been associated with negative effects, such as later referrals or less conformity to guidelines.^32–35^ In addition, seeing the same doctor over time does not guarantee a good doctor–patient relationship.^29,36^ It may also be too simplistic to assume that continuity is always important for the patient with some prioritising an urgent appointment or specific expertise.^33,37,38^

General practice is changing, as evidenced in the 2019 *NHS Long Term plan*. This document has a strong focus on increasing patient access to primary care with no specific references to improving continuity in general practice.^39^ This could perhaps represent a shift in government priorities, although by increasing the workforce and services available in primary care, continuity could be affected in a number of ways.^40^ The Nuffield Trust produced a report describing how structural changes such as personal lists, longer consultations, and broadening the workforce in primary care could achieve both targets of increasing access and continuity of care.^40^ If both these aims are achieved it will be interesting to observe any effect on frequent attendance in the future.

## Trial registration number

ISRCTN: 62939408
